# Assessment of the physicochemical and sensory characteristics of fermented camel milk fortified with *Cordia myxa* and its biological effects against oxidative stress and hyperlipidemia in rats

**DOI:** 10.3389/fnut.2023.1130224

**Published:** 2023-05-09

**Authors:** El Sayed Hassan Atwaa, Magdy Ramadan Shahein, Enrique Raya-Álvarez, El Sayed Abd El-Sattar, Moustafa A. A. Hassan, Madeha Ahmed Hashim, Naief Dahran, Manal F. El-Khadragy, Ahmad Agil, Ehab Kotb Elmahallawy

**Affiliations:** ^1^Department of Food Science, Faculty of Agriculture, Zagazig University, Zagazig, Egypt; ^2^Department of Food Science and Technology, Faculty of Agriculture, Tanta University, Tanta, Egypt; ^3^Department of Rheumatology, Hospital Universitario San Cecilio, Granada, Spain; ^4^Department of Food and Dairy Technology, Faculty of Technology and Development, Zagazig University, Zagazig, Egypt; ^5^Department of Food Science, Faculty of Agriculture, Ain Shams University, Cairo, Egypt; ^6^Department of Histology, Faculty of Veterinary Medicine, Sohag University, Sohag, Egypt; ^7^Department of Anatomy, Faculty of Medicine, University of Jeddah, Jeddah, Saudi Arabia; ^8^Department of Biology, College of Science, Princess Nourah bint Abdulrahman University, Riyadh, Saudi Arabia; ^9^Department of Pharmacology, Biohealth Institute Granada (IBs Granada) and Neuroscience Institute, School of Medicine, University of Granada, Granada, Spain; ^10^Department of Zoonoses, Faculty of Veterinary Medicine, Sohag University, Sohag, Egypt

**Keywords:** *Cordia myxa*, camel milk, lipid profile, biochemical markers, kidney function, liver function

## Abstract

Natural feed additives and their potential benefits in production of safe and highly nutritious food have gained the attention of many researchers the last decades. *Cordia myxa* is a nutrient-dense food with various health benefits. Despite this fact, very limited studied investigated the physicochemical and sensory impacts of incorporation of fermented camel milk with *Cordia myxa* and its biological effects. The current study aimed to assess the physical, chemical, and sensory characteristics of fermented camel milk (FCM) fortified with 5, 10, and 15% *Cordia myxa* pulp. The study demonstrated that fortification of camel milk efficiently enhanced protein, total solids, ash, fiber, phenolic substance, and antioxidant activity. When compared to other treatments, FCM supplemented with 10% *Cordia myxa* pulp had the best sensory features. In addition, FCM fortified with 10% *Cordia myxa* pulp was investigated as a potential inhibitor of hypercholesterolemia agents in obese rats. Thirty-two male Wistar rats were split into two main groups including normal pellet group (*n* = 8) served as negative control group (G1) and a group of hyperlipidemic animals (*n* = 24) were feed on a high-fat diet (HFD). Hyperlipidemic rats group (*n* = 24) were then divided into three subgroups (8 per each); second group or positive control (G2) which include hyperlipidemic rats received distilled water (1 mL/day), the third group (G3) involved hyperlipidemic rats feed on FCM (10 g/day) and the fourth group (G4) included hyperlipidemic animals feed on 10 g/day FCM fortified with 10% of *Cordia myxa* pulp by oral treatment via an intestinal tube for another 4 weeks. In contrast to the positive control group, G4 treated with *Cordia myxa* showed a substantial decrease in malondialdehyde, LDL, cholesterol, triglycerides, AST, ALT, creatinine, and urea levels, while a significant increase in HDL, albumin, and total protein concentrations. The number of large adipocytes decreased while the number of small adipocytes increased after consumption of fortified FCM. The results indicated that fermented milk fortified with *Cordia myxa* pulp improved the functions of the liver and kidney in hyperlipidemic rats. These results demonstrated the protective effects of camel milk and *Cordia myxa* pulp against hyperlipidemia in rats.

## 1. Introduction

Cardiovascular diseases (CVDs) continue to be the world’s leading cause of death (1). According to previous studies (2, 3), the development of atherosclerotic plaque is an inflammatory process in the endothelial vessel wall linked to retained low-density lipoprotein (LDL). Hypercholesterolemia, or elevated plasma cholesterol, is a significant cardiovascular risk factor. Changing lifestyles and using medications are two ways to treat high plasma LDL cholesterol (4, 5). Despite the fact that lipid-lowering medication is unquestionably effective in lowering the frequency of cardiovascular events, a sizable portion of people rejects pharmacological therapy. The potential for nutraceuticals as an alternate strategy and method of treatment for hypercholesterolemia exists (6). Additionally, a balanced diet and dietary supplements are advised by the European Society of Cardiology, the International Lipid Expert Panel, the European Atherosclerosis Society, and other guidelines to enhance lipid profiles (7, 8). Therefore, it is not surprising to mention that many previous studies revealed the potential benefits of incorporating several natural substances into several dairy products as potential functions foods (9–17). “Nutraceuticals” is a wide umbrella term that is used to explain any product stemmed from food bases with further health benefits in addition to the essential nutritional value discovered in foods. Nutraceuticals that decrease cholesterol are sometimes recommended. In order to treat hypercholesterolemia and maintain the physiological levels of plasma cholesterol, several meta-analyses demonstrated the effectiveness of common nutraceuticals with various mechanisms of action (such as fiber, polyphenol, and flavonoids) (18, 19).

Many plants, including *Cordia myxa*, known as Sebastian plum, soapberry, or mokhate in Egypt, have potent antioxidant qualities. One kernel seed plus a pulpy portion make up the fruit. A number of pathological conditions, including anemia, rheumatism, gastric pain, impotence, diarrhea, mouth ulcers, bronchitis, asthma, and dental caries, were previously treated using *Cordia myxa* (20, 21). Fruits have hepatoprotective, wound-healing, anti-ulcer, and anthelmintic properties, as well as hypolipidemic and anti-diabetic properties (22–24). Polyphenols, flavonoids, alkaloids, and mucilage are just a few of the essential substances found in *Cordia myxa* fruit (25). The viscid layer aqueous extract contains the major polyphenols quercetin, rosmarinic acid, caffeic acid, kaempferol, chlorogenic acid, and rutin (26–28). According to the nutritional needs of the infant, mammalian milk is quite nutritious. Even though camel milk (*Camelus dromedarius* L.) is the most popular milk in the Arab Gulf nations, it only has a minor global impact. However, the origin of the milk being investigated, transitory or physiological fluctuations, race, keeping circumstances (particularly feeding), biological stage or animal health state, and analytical faults all have an impact on the data available in the literature concerning camel milk structure. Camel milk varies greatly from cow’s milk in terms of its low coagulability and chemical composition (29, 30). It has repeatedly been proposed that camel milk has health benefits for people with metabolic syndrome, and this condition is characterized by abdominal obesity, dyslipoproteinemia (lower HDL and higher VLDL), and hypertension (30, 31). According to published research, the protein hydrolysates of camel milk have significant bio-functional properties including antioxidant, anti-diabetic, anticancer, antiallergic, hepatoprotective, anti-inflammatory, antimicrobial, angiotensin-converting enzyme inhibitory, antiradical, and anti-autism activities (32, 33). Some antioxidant enzymes, micronutrients, and protective proteins are the chemicals that have the biggest biological effects on physiological processes (34). Adults with hypertension and diabetes benefit from frequent use of camel milk over the short and long term because it inhibits oxidative and inflammatory stresses (35). Previous work reported (36) significant variations in the protein content and molecular properties of camel milk proteins. Inhibiting the enzymes that cause hypertension, having antibacterial and antioxidant properties, and having anti hyperlipidemic effects are all potential health benefits (37). The current study’s main objective is to investigate the hepatoprotective health effects of fermented camel milk (FCM) and *Cordia myxa* pulp in hyperlipidemic rats with HF-diet-induced obesity, as well as on lipid profiles, biochemical markers, and histopathological features in rats with hypercholesterolemia.

## 2. Materials and methods

### 2.1. Materials and reagents

The Desert Research Center in Dokki, Egypt, provided fresh camel milk. On August 2022, fruits were harvested from Cordia trees in the Sharkia region of Egypt. Agric located the tree in the Orman Botanical Garden in Giza, Egypt. Reagents chemicals were purchased from Sigma-Aldrich (Cairo, Egypt). Gallic acid and 1,1-diphenyl-2-picrylhydrazyl (DPPH) were also provided by Sigma (St. Louis, MO, USA). Human, Gesellschaft für Biochemica and Diagnostica mbH, Wiesbaden, Germany, provided the equipment for detecting total protein, serum albumin, total cholesterol, triglycerides, HDL, GSH-Rd, AST, ALT, SOD, GSH-Px, creatinine, and urea. Chr. Hansen (Hrsholm, Denmark) provided the lyophilized starter culture ABT-5, which contains *Streptococcus thermophiles*, *Lactobacillus acidophilus*, and *Bifidobacterium bifidum*.

### 2.2. Preparation of *Cordia myxa* pulp (CMP)

The seeds of the fully developed *Cordia myxa* fruits were removed after they had been meticulously cleaned and washed several times under running water. The deseeded fruits were heated to 83°C in a steam-jacketed jar (Width: 27 (in); Height: 38 5/8 (in); Depth: 38 1/2) for 2 min as a pasteurizer for the fruits and to improve their texture, and then cooled to room temperature at 30°C. To obtain pulp with a uniform texture, the pulp was crushed at high speed using an electric mixer (Braun, Germany). We used the prepared fruit homogenates right away.

### 2.3. Fermented camel milk manufacture

The procedures for making FCM presented by Tamime and Robinson (38). For 30 min, raw camel milk (3 L for each treatment) was heated to 85°C before being cooled to 42°C in an ice bath then, placed in a 5-liter durable glass universal jar equipped with a plastic cover that ensures airtight closure and for conduction the fermentation process. ABF-5 (0.02%, 50 units) lyophilized starter culture including *Streptococcus thermophilus*, *Lactobacillus acidophilus*, and *Bifidobacterium bifidum* (starting count of log 10^7^ CFU/ml for each of the bacteria strains present) was then added. Then, it was incubated at 42°C for approximately 12 h to produce a firm curd, mixed with a mixer, and then divided into 4 equal sections. The first part was used as the control. The remaining three halves were thoroughly combined with 5, 10, and 15% pulp from *Cordia myxa*. Then, 100 mL plastic bottles with caps were filled with the FCM treatments, which were then kept at 4°C and examined 1 day after production. Each analysis was performed twice, and the experiments were repeated in triplicate.

### 2.4. Physicochemical, chemical analysis, and sensory assessment of fermented camel milk treatments

The chemical analysis was done on various samples of FCM (4 samples and each sample has three replicates), camel milk (one sample with three replicates) and *Cordia myxa* pulp (One sample with three replicates) to assess the pH, protein, ash, titratable acidity, moisture, fiber, and fat contents according to AOAC procedures (39). The total phenolic content was determined using a spectrophotometer and the Folin–Ciocalteu assays (Secomam, France) (40). The antioxidant activity was assessed via DPPH assay (41). *AOA (%) = 1- Abs sample _ Abs blank/Abs control ×100* was used to calculate the antioxidant activity percentag. Using the scoring provided by Tamime and Robinson (38), and modified as described elsewhere (12), this step of sensory investigations was completed by a total of 20 trained and untrained panelists. The assessment crew was comprised of both untrained and trained panelists. Panelists were instructed to assess the flavor (50 points), Consistency (30 points), Appearance (20 points) and total scores (100 points). The sensory evaluation of the different descriptors relied on the pre-selected descriptors: appearance (wheying-off, white color, reddish color), consistency (ropy, uniform coagulum), flavor (sweetness, acidity, bitterness), and total score (the sum of all the character results).

### 2.5. Experimental design

The experimental protocol of this study was approved by the research ethical committee of the Faculty of Science, Tanta University and the institutional Review Board Number IACUC-SCI-TU-0300. A total of 32 male Wistar rats (200 ± 10 g) were provided to the Agricultural Research Center (Giza, Egypt). All animals were maintained in a room with free access to food and water, controlled lighting (12 h of light and 12 h of darkness), under a relative humidity of 40–60% and an ambient temperature of 22 ± 2°C. All animals had unrestricted access to regular food in compliance with AIN-93 guidelines (42). The rats were split into two main groups and given either normal pellet to serve as control negative group (*n* = 8) and another group of animals (*n* = 24) received high-fat diet (HFD) consisting of 1% pure cholesterol, 31.70 g animal fat, 67 g regular food, and 0.30% bile acid as reported elsewhere (43). This later group of animals was then divided into three subgroups (*n* = 8 animal per group) and feed on this high-fat diet for 4 weeks to promote obesity. This was done after a one-week acclimation phase. Obese rats were subjected to receive a distilled water (1 mL/day) to serve as positive control group (G2), G3 received FCM (10 g/day) and G4 that received 10 g/day FCM fortified with 10% of *Cordia myxa* pulp by oral treatment via an intestinal tube for another 4 weeks. At the end of study period (8 weeks), rats were fasted overnight and euthanized under full anesthesia by intraperitoneal injection of ketamine 90 mg/kg and xylazine 5 mg/kg. After gently separating the abdomen skin from the thoracic cavity, blood was drawn from the posterior vena cava and put into a serum separator tube. Sera were then obtained by centrifuging the collected blood at 3000 rpm for 10 min. The serum samples were then stored at −20°C until analysis.

### 2.6. Biological analysis

Serum levels of total cholesterol, HDL, and triglycerides were estimated (44). As detailed earlier, serum albumin, liver enzymes, and total protein levels were also determined as mentioned elsewhere (45). As markers of renal function, urea and creatinine were assessed (46). Serum malondialdehyde MDA, and lipid peroxides were assessed (47). The Superoxide dismutase (SOD) and total antioxidant capacity (TAC) activities were tested (48, 49), while glutathione (GSH) was measured as reported elsewhere (50).

### 2.7. Measurement of adipocytes

All of the rats were euthanized, at the end of 8 weeks, for histological analysis. The adipose tissue collected in visceral fat surrounding the kidney such as mesenteric, retroperitoneal and epididymal white adipose tissue. The liver and adipose tissues were embedded in paraffin wax, fixed in a 4% formaldehyde solution, and stained with hematoxylin–eosin (H&E) (51). Microscopy was used to observe and photograph histopathological changes (Nikon Ts2R, Japan). With the use of ImageJ software, the adipocyte sizes were calculated (NIH, Bethesda, Rockville, MD, USA).

### 2.8. Morphometric measurement

Hepatic tissue samples were assessed and scored semiquantitatively based on the visual field inspection of a minimum of 10 sections from each group. Photographs were then taken at a magnification of 40×. Analysis of organ histopathology was also performed by assigning a score depending on the degree of fatty degeneration (Vacuolar degeneration): 0 = no lesions; 1 = mild (1 to 25%); 2 = moderate, (26–45%); 3 = severe (>45%) as described previously (52–55).

### 2.9. Statistical analysis

The findings of all experiments and related analyses, carried out in triplicate, were reported as means and standard deviations. The variation across groups was determined using a one-way ANOVA with a significance criterion of *p ≤* 0.05 followed by the least significant difference (LSD) test. Statistica 12.5 software was used to carry out the analysis (Stat Soft Inc., Tulsa, OK, USA).

## 3. Results and discussion

### 3.1. Chemical and phytochemical properties of camel milk and *Cordia myxa* pulp

The phytochemical and chemical characteristics of camel milk and CMP are displayed in Table 1. Camel milk contained 12.96, 3.55, 4.66, 0.84, and 0.00 g/100 g, respectively, of total solids (TS), protein, fat, ash, and fiber. These findings were in line with previous work (56) that revealed that camel milk contained 4.24, 3.55, 0.87, 5.65, and 14.31 g/100 g of lactose, protein, ash, fat, and TS, respectively. Additionally, Karaman et al. (57) discovered that camel milk contained 3.10, 11.83, 0.83, and 3.28 g/100 g, respectively, of protein, TS, ash, and fat. This vast diversity in milk content may be influenced by several variables, including the animal’s health, genetic characteristics, the lactation stage, and environmental variables (58). The chemical composition of CMP was as follows: protein, TS, fat, ash, and fiber contents were, respectively, 1.69, 18.3, 1.12, 0.47, and 8.89 g/100 g. The TS, protein, fat, ash, and fiber contents of CMP ranged from 18.00 to 20.0, 8.68 to 9.41, 0.57 to 2.83, 6.33 to 7.93, and 10.17 to 50.5 g/100 g, respectively. According to several previous works (59, 60), total phenolic content (TPC) and DPPH% of camel milk were 98.0 mg/100 g and 19.52%, respectively. These findings agreed with El-Fattah et al. (61), who reported that camel milk’s DPPH% inhibition was 18.57%, whereas CMP’s TPC and DPPH% were 682.70 mg/100 g and 80.70%, respectively. Several previous reports (25, 59, 62) explained that fruit from *Cordia myxa* is rich in bioactive compounds such as flavonoids and phenolics.

**Table 1:** Chemical composition and phytochemical properties of camel milk and *Cordia myxa* pulp (CMP).

Components (%)	Camel milk	*Cordia myxa* pulp (CMP)
Total solids	12.96 ± 0.22	18.3 ± 0.94
Protein	3.55 ± 0.09	1.69 ± 0.08
Fat	4.64 ± 0.12	0.47 ± 0.02
Ash	0.84 ± 0.01	1.12 ± 0.16
Fiber	0.00	8.89 ± 0.86
Carbohydrate	4.11 ± 0.10	15.02 ± 1.66
Phytochemical properties		
TPC (mg/100 g)	98.0 ± 5.12	682.70 ± 20.14
DPPH %	19.52 ± 0.04	80.70 ± 4.05

### 3.2. Physicochemical, phytochemical and sensory properties of fermented camel milk supplemented with CMP

Table 2 displays the impact of CMP addition on the physicochemical, phytochemical, and sensory aspects of camel milk. Due to the addition of CMP, the TS, fiber, and ash contents of camel milk increased considerably (*p* ≤ 0.05) from 13.24, 0.00, and 0.92 in the control samples to 15.94, 1.32, and 1.09 in the samples fortified with 15% CMP (T3). The high TS, ash, and fiber contents CMP compared to camel milk (28). On contrary, due to the low protein and fat concentrations of CMP, the inclusion of CMP in fermented camel milk (FCM) in varying proportions had no influence on the protein and fat contents. These findings corroborated with several previous works (30, 63, 64) that discovered that camel milk supplemented with avocado, kiwi, Sukkari dates, and sidr fruit pulp had higher TS, ash, and dietary fiber concentrations when compared to regular FCM. Regarding TA, Table 2 demonstrates that increasing the fortification percentage greatly reduced (*p* ≤ 0.05) the acidity of FCM fortified with CMP. This might be explained by the antimicrobial substance’s CMP content, which might reduce starting culture viability (26–28). In comparison to fortified treatments, plain FCM had a greater TA value. In contrast to plain FCM, the pH values of CMP-enhanced treatments rose according to the degree of fortification. Similar findings were made in an earlier study (30), which discovered boosting the pH and lowering the TA values of FCM by adding sidr fruit pulp. Another study (65) revealed that camel milk yogurt’s pH and TA values increased when it was fortified with monk fruit sweetener. The pulp-induced high-viscosity conditions greatly raised (*p* ≤ 0.05) the viscosity values of the treatments enhanced with CMP, and this rise was proportionate to the fortification ratios (25). Similarly, earlier investigations (30, 65) found that the viscosity of camel milk yoghurt increased when sidr fruit pulp or monk fruit sweetness was added.

**Table 2:** Chemical composition, physicochemical, phytochemical, and sensory properties of FCM supplemented with *Cordia myxa* fruit pulp.

Item	Treatments				
	C	T1	T2	T3	
Chemical composition%					LSD
Total solids	13.24 ± 0.66 ^d^	14.02 ± 0.74 ^c^	15.04 ± 0.64 ^b^	15.94 ± 0.86 ^a^	0.2278
Protein	3.68 ± 0.12 ^a^	3.74 ± 0. 07 ^a^	3.82 ± 0.18 ^a^	3.92 ± 0.14 ^a^	0.2139
Fat	4.75 ± 0.14 ^a^	4.78 ± 0.09 ^a^	4.80 ± 0.14 ^a^	4.82 ± 0.12 ^a^	0.2194
Ash	0.92 ± 0.08 ^d^	0.98 ± 0.04 ^c^	1.03 ± 0.05 ^b^	1.09 ± 0.04 ^a^	0.0898
Fiber	0.0 ± 0.00 ^d^	0.44 ± 0.03^c^	0.90 ± 0.02 ^b^	1.32 ± 0.05 ^a^	0.0330
Physicochemical properties					
Acidity%	0.90 ± 0.04 ^a^	0.87 ± 0.03 ^b^	0.78 ± 0.03 ^c^	0.75 ± 0.02 ^d^	0.0688
pH values	4.70 ± 0.03 ^d^	4.73 ± 0.01 ^c^	4.76 ± 0.02 ^b^	4.80 ± 0.04 ^a^	0.0872
Viscosity (cP)	2010 ± 74 ^d^	2670 ± 90 ^c^	3050 ± 96 ^b^	3640 ± 85 ^a^	46.810
Phytochemical properties					
TPC (mg/g)	114.0 ± 7.12^d^	145.0 ± 6.40^c^	186.0 ± 8.20 ^b^	224 ± 5.50 ^a^	11.856
DPPH %	20.14 ± 0.94 ^d^	23.36 ± 1.33 ^c^	27.12 ± 1.22 ^b^	31.40 ± 1.14 ^a^	5.1623
Sensory properties					
Flavor (50)	38.20 ± 1.72 ^c^	40.30 ± 1.84 ^b^	42.50 ± 1.18 ^a^	42.70 ± 1.36 ^a^	0.5829
Consistency (30)	21.80 ± 1.14 ^d^	24.20 ± 1.34 ^c^	27.60 ± 1.18^a^	25.70 ± 1.42 ^b^	0.4897
Appearance (20)	14.30 ± 0.72 ^d^	15.10 ± 0.84 ^c^	16.40 ± 0.92 ^a^	14.80 ± 0.96 ^b^	0.4617
Total scores (100)	74.30 ± 2.14 ^d^	79.6 ± 2.22 ^c^	84.5 ± 2.16 ^a^	83.2 ± 2.36 ^b^	0.8162

The small letters a, b, c, etc. in the same column vary significantly at (*p* ≤ 0.05). LSD, Least significant difference. Camel milk fermented as a control (C). T1, FCM with 5% CMP added; T2, FCM prepared with 10% CMP and camel milk; T3, FCM produced from FCM and 15% CMP.

In Table 2, as the supplementation ratio rose, the TPC and DPPH% of FCM supplemented with CMP increased considerably (*p* ≤ 0.05) compared to FCM without CMP. The high TPC of CMP in comparison to that of camel milk may be the cause (28, 63). The TPC and DPPH% of yoghurt were observed to rise when sidr fruit pulp was added to camel milk in a previous study (30). The TPC and DPPH% of FCM were also observed to increase when kiwi and avocado were added in a previous study (63). The sensory qualities of the resulting FCM greatly improved (*p* ≤ 0.05) when CMP was added to camel milk, as indicated in Table 2, when compared to plain FCM, especially consistency and flavor. Additionally, this enhancement decreased as the supplementation ratio reached 15%. Lower sensory property scores were obtained for the plain FCM. This might be brought on by the inferior flavor, weak body, and texture of camel milk curd. The lack of β-LG in camel milk, the large casein micelles, the relative distribution of casein fractions, and the tiny size of the camel fat globules all led to the watery and fragile quality and poor structure of the plain FCM (64). These findings were in line with several earlier studies (30, 64), which showed that fortifying camel milk with Sukkari dates or sidr fruit pulp improved its sensory qualities.

### 3.3. Effects of fermented camel milk supplemented with CMP on hyperlipidemic rats’ final weight and body weight gain (BWG)

Table 3 displays the effects of FCM fortified with CMP on the body weight gain (BWG) and final weight of hyperlipidemic rats. The present findings revealed that the initial weights of non-treated non-hyperlipidemic rats (negative control), hyperlipidemic rats (positive control), hyperlipidemic rats given FCM supplemented with 10% CMP, and hyperlipidemic rats were given FCM were 204.8, 205.2, 206.2, and 207.3 g, respectively. The treatments had a significant (*p* ≤ 0.05) impact on the FW and BWG of the rats. The lower BWG values (31.81%) were produced in hyperlipidemic rats receiving 10 g/day of FCM supplemented with 10% CMP, followed by 33.93% in rats receiving only 10 g/day of FCM. In contrast, the animals administered FCM supplemented with 10% CMP had lower FW (334.3 g) and BWG (by 38.61%) than the positive control group. The mean food intakes were similar in all experimental groups. Along with a reduction in relative weight, the high CMP content from bioactive components, including vitamins, flavonoids, phenolic acids, and minerals, may have played a role in the drop in blood fat levels in rats. These bioactive components may be responsible for enhancing the BWG and final weight of hyperlipidemic rats fed FCM augmented with 10% CMP (28). Furthermore, camel milk’s high mineral and vitamin C content may serve as an antioxidant that fights free radicals (34). Accordingly, *Cordia myxa* fruit improved the nutritional condition and decreased the BWG of hyperlipidemic rats, according to El-Newary et al., 2018 (25). In contrast, in another study (66), camel milk curd improved the nutritional condition and reduced BWG in hyperlipidemic rats. Compared to other hyperlipidemic rats, hyperlipidemic rats given FCM and 10% CMP supplementation revealed the finest results in terms of FW and BWG.

**Table 3:** Final weight and body weight gain of hyperlipidemic rats treated with FCM containing CMP.

Group	Parameters			
	Initial weight (g)	Final weight (g)	B W G %	Food intake (g/day)
Group (1)	204.8 ± 4.2 ^a^	292.2 ± 3.5^a^	29.91 ± 1.9 ^a^	20.86 ± 0.9^a^
Group (2)	205.2 ± 3.4 ^a^	334.3 ± 4.2 ^d^	38.61 ± 1.7 ^d^	20.12 ± 0.96^a^
Group (3)	207.3 ± 3.8 ^a^	313.8 ± 5.4 ^c^	33.93 ± 1.4 ^c^	19.94 ± 0.88^a^
Group (4)	206.2 ± 5.2 ^a^	302.4 ± 3.8 ^b^	31.81 ± 1.6 ^b^	19.78 ± 1.02^a^
LSD	0.6270	3.9987	1.4138	0.2184

Mean values of eight rats ± SD. a, b, c…. of the small letters in in the same column vary significantly at (*p* ≤ 0.05). LSD, Least significant difference. Group (1) rats without treatment for hyperlipidemia (negative control). (2) rats with hyperlipidemia (positive control). Rats with hyperlipidemia that were treated with FCM (Group 3) Group 4 hyperlipidemic rats given FCM with 10% CMP.

### 3.4. Effects of CMP-supplemented fermented camel milk on hyperlipidemic rats’ blood lipid profiles

In Table 4, the group of hyperlipidemia rats that were fed FCM fortified with 10% CMP showed a lower level of total cholesterol (77.80 mg/dL) compared to the positive control group (105.55 mg/dL). In addition, the positive control group showed higher levels of triglycerides and LDL, which was 129.38 and 51.52 mg/dL, respectively, compared to the other groups. Moreover, hyperlipidemia rats that were given FCM and FCM fortified with CMP showed lower triglyceride and LDL levels of 98.11, 91.07, 39.60, and 34.08 mg/dL, respectively. The obese groups treated with both FCM and FCM fortified with CMP showed higher HDL-C values than the positive control group (28.15 mg/dL). In this concern, rats were given CMP-supplemented FCM showed a significant (*p* ≤ 0.05) rise in HDL levels (37.12 mg/dL). The large number of elements in CMP can interact with degrading enzymes of lipids to emulsify fat, hydrolyze it, and dissolve micelles, leading to absorption (28), which might be caused by the cause of the antihyperlipidemic effects seen in CMP. These components include minerals, vitamins, polyphenols, and flavonoids. The antihyperlipidemic action may also be induced by snatching free radicals, sustaining HDL-binding paroxonase activity by oxidized metal ions chelation and LDL oxidation inhibition (67). Additionally, camel milk’s high insulin content has been shown to activate the lipoprotein lipase enzyme (68). Additionally, the high content of minerals in camel milk (zinc, sodium, magnesium, copper, and potassium) along with vitamin C consumption may combine to neutralize free radicals (30). Several previous studies (25, 66) also discovered that the fruit of the *Cordia myxa* plant or FCM had a hypocholesterolemic impact. Several kinds of antioxidants, including polyphenols and glucosinolates, have been shown to have beneficial effects on the reversal of fatty liver. Although the exact mechanism of action is yet unclear, in some circumstances an indirect contact with mitochondrial metabolism is anticipated, which could lead to the creation of cutting-edge therapeutic strategies for treating fatty liver (69). It is also noteworthy to mention that the polyphenols alter signaling pathways that control adipogenesis, antioxidant defenses, and anti-inflammatory responses, such as AMP-activated protein kinase, peroxisome proliferator activated receptor alpha, sterol regulatory element binding protein-1c, uncoupling proteins, and nuclear factor kappa B (70).

**Table 4:** Effect of FCM supplemented with CMP on the serum lipid profile in hyperlipidemic rats.

Groups	Parameters			
	Total cholesterol (TC) (mg/dL)	Triglycerides (TG) (mg/dL)	HDL (mg/dL)	LDL (mg/dL)
Group (1)	77.80 ± 7.34^d^	85.77 ± 9.49^d^	39.57 ± 3.71^a^	21.07 ± 1.04^d^
Group (2)	105.55 ± 12.75^a^	129.38 ± 11.61^a^	28.15 ± 2.43 ^d^	51.52 ± 3.92 ^a^
Group (3)	96.35 ± 8.62^b^	98.11 ± 10.22^b^	37.12 ± 2.61^b^	39.60 ± 2.12^b^
Group (4)	88.31 ± 7.43^c^	91.07 ± 7.21^c^	36.02 ± 1.75^c^	34.08 ± 2.66^c^
LSD	7.0688	3.5579	1.6325	2.9430

Mean values of eightsix rats ± SD. a, b, c…. of the small letters in the same column vary significantly at (*p* ≤ 0.05). LSD, Least significant difference.

### 3.5. Effects of CMP – supplemented fermented camel milk on liver function metrics in hyperlipidemic rats

Table 5 presents the effect of FCM fortified with CMP on measures of liver function in hyperlipidemia rats. Obese rats (positive control group) showed higher levels of ALT and AST in plasma (*p* ≤ 0.05) and lower levels of total albumin and protein than the obese rats fed FCM fortified with CMP. However, compared to the positive control group, plasma levels of ALT and AST have decreased (*p* ≤ 0.05), while total protein and albumin have increased. The high flavonoid and phenolic acid contents of CMP exert antioxidant properties by grabbing free radicals (25, 62), which are responsible for the hepatoprotective actions of CMP. Additionally, camel milk’s high vitamin C content may function as an antioxidant, and thereby, this reduces aminotransferase enzymes (30). These findings corroborated several previous works (59, 66) that discovered hepatoprotective benefits of FCM or *Cordia myxa* fruit. Comparatively to other hyperlipidemic rats, hyperlipidemic rats given FCM supplemented with 10% CMP demonstrated enhanced liver function.

**Table 5:** Effect of FCM supplemented with CMP on liver function parameters in hyperlipidemic rats.

Group	Aspartate aminotransferase (AST U/L)	Alanine aminotransferase (ALT U/L)	Total protein (g/dL)	Total albumin (g/dL)
Group (1)	47.66 ± 2.32^d^	32.26 ± 1.28^cd^	6.98 ± 0.52 ^a^	4.06 ± 0.45 ^a^
Group (2)	68.71 ± 2.88^a^	49.09 ± 2.24^a^	5.04 ± 0.28 ^c^	2.94 ± 0.54 ^c^
Group (3)	53.15 ± 2.05^b^	38.00 ± 1.14^b^	5.82 ± 0.60 ^b^	3.50 ± 0.28 ^b^
Group (4)	51.45 ± 1.45^c^	33.00 ± 1.18^c^	6.24 ± 0.42 ^ab^	3.90 ± 0.38 ^ab^
LSD	4.2413	1.6833	0.0979	0.2013

Mean values of eightsix rats ± SD. a, b, c…. of the small letters in the same column vary significantly at (*p* ≤ 0.05). LSD, Least significant difference.

### 3.6. Effects of CMP – supplemented fermented camel milk on kidney function metrics in hyperlipidemic rats

Plasma creatinine and urea concentrations significantly (*p* ≤ 0.05) increased in the positive control group. In addition, these measured values significantly dropped in the hyperlipidemic groups administered FCM and FCM supplemented with CMP (Table 6). In addition, the positive control group exhibited a statistically significant (*p* ≤ 0.05) increase in lipid peroxidation, as shown by its raised MDA levels in compared to the treated hyperlipidemic groups. The hyperlipidemic group that received FCM combined with CMP had a substantial (*p* ≤ 0.05) decrease in MDA levels. Natural antioxidants can prevent the oxidative processes, high cytotoxicity, and inhibitory effects that might result from the development and accumulation of MDA (71). This remarkable shift may be attributed to the high concentration of bioactive components in CMP, including minerals, vitamins, flavonoids, and phenolics, which function as superoxide scavengers and limit the formation of uric acid and reactive oxygen species (25, 66). Additionally, camel milk possesses nephroprotective qualities as a result of its antioxidants, which is associated with its high vitamin C and mineral contents (34). Several previous works (30, 59) found that *Cordia myxa* fruit or FCM had nephroprotective effects, and these findings corroborated their findings. Comparatively to other hyperlipidemic rats, hyperlipidemic rats fed 10% CMP-supplemented FCM had the greatest results for serum renal function.

**Table 6:** Effect of FCM supplemented with CMP on kidney function parameters in hyperlipidemic rats.

Group	Creatinin (mg/dL)	Urea (mg/dL)	Malondialdehyde (MDA) (μmol/L)
Group (1)	0.85 ± 0.02^d^	14.80 ± 1.22^d^	37.46 ± 3.72^d^
Group (2)	1.74 ± 0.21^a^	36.10 ± 3.02^a^	75.31 ± 5.30^a^
Group (3)	1.03 ± 0.52^b^	24.05 ± 2.70^b^	52.02 ± 3.11^b^
Group (4)	0.94 ± 0.11^c^	17.85 ± 2.14^c^	46.35 ± 4.29^c^
LSD	0.0163	1.7658	4.8974

Mean values of eight rats ± SD. a, b, c…. of the small letters in the same column are significantly different at (*p* ≤ 0.05). LSD, Least significant difference.

### 3.7. Effects of fermented camel milk supplemented with CMP on serum levels of the enzymes GSH, SOD, and TAC in hyperlipidemic rats

The information in Table 7 demonstrates that the hyperlipidemic rats’ serum TAC, GSH-px, and SOD levels were considerably lower than those of the control rats. In comparison to the positive control group, hyperlipidemic rats which consumed FCM and FCM supplemented with CMP had significantly (*p* ≤ 0.05) greater blood TAC, GSH-px, and SOD levels. These findings indicated that the FCM offered antioxidative and health advantages for livers recovering from damage caused by a high-fat diet. The activities of these enzymes increased significantly, indicating that camel milk and CMP have antihepatotoxic and antioxidant properties, as bioactive ingredients, ascorbic acid and phenolic chemicals in camel milk and CMP may neutralize free radicals, active oxygen species, and reduce oxidative stress and liver inflammation (25, 34). These findings were in line with a variety of earlier studies (25, 30) that found that *Cordia myxa* fruit or FCM had antioxidant effects. Comparatively to other hyperlipidemic rats, hyperlipidemic rats administered FCM augmented with 10% CMP generated the highest serum TAC, GSH-px, and SOD levels.

**Table 7:** Effect of FCM fortified with CMP on antioxidant markers in hyperlipidemic rats.

Groups	TAC (μmol/L)	SOD (U/g Hb)	GSH-px (U/g Hb)
Group (1)	820 ± 12^a^	6.22 ± 0.92^a^	25.18 ± 0.86^a^
Group (2)	590 ± 18^d^	4.18 ± 0.76^c^	17.22 ± 0.74^d^
Group (3)	720 ± 21^c^	5.24 ± 0.82^bc^	20.34 ± 0.80^c^
Group (4)	790 ± 20^b^	5.90 ± 0.65^b^	23.28 ± 0.92^b^
LSD	87.911	0.5831	0.2196

Mean values of eight rats ± SD. a, b, c…. of the small letters in the same column are significantly different at (*p* ≤ 0.05). LSD, Least significant difference.

### 3.8. Effects of fermented camel milk supplemented with CMP on adipocyte volume in hyperlipidemic rats and adipose tissue weight

**Figure 1:**
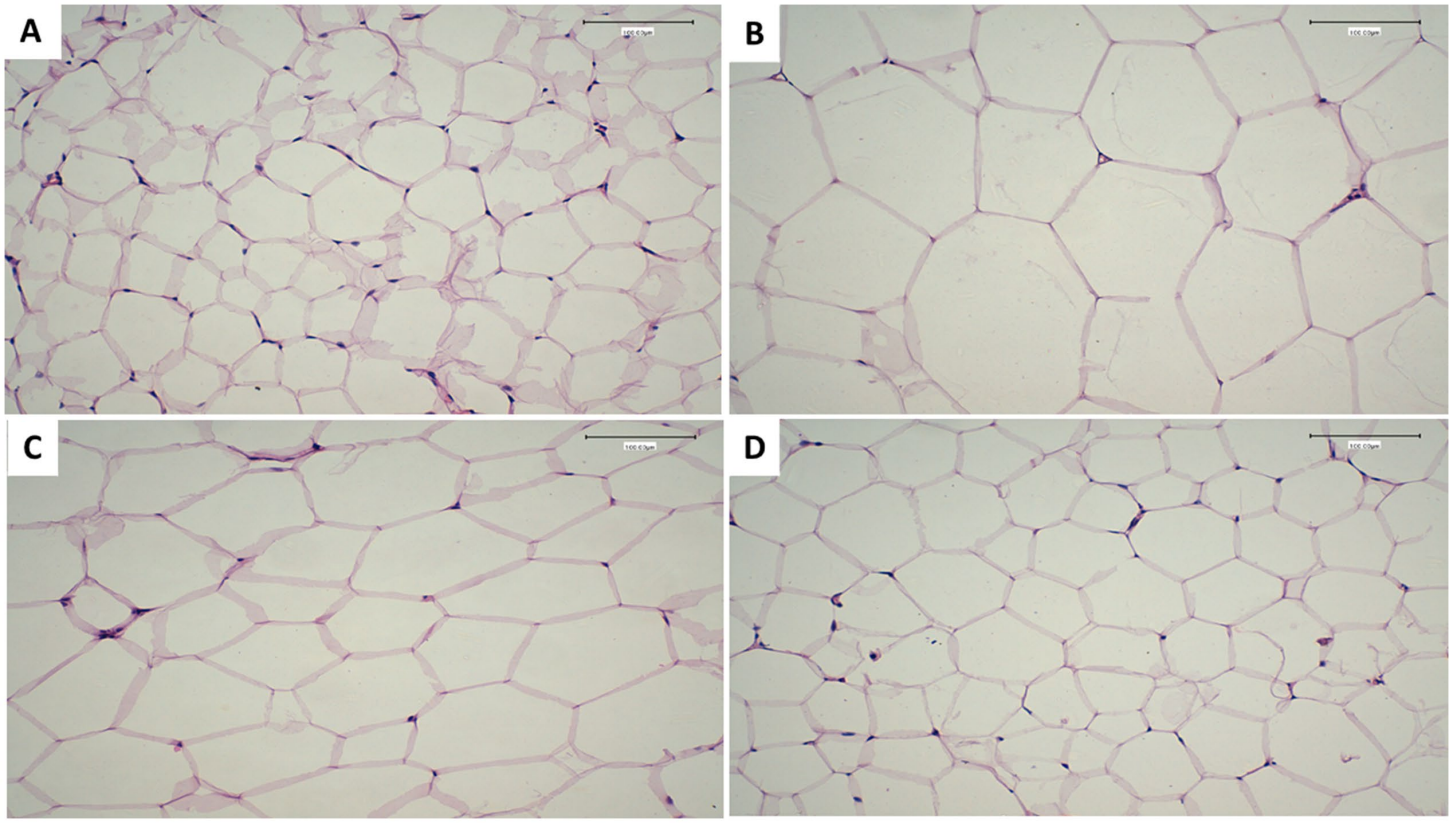
Effects of FCM supplemented with CMP on adipocyte cells showing adipocytes in paraffin sections showing. **(A)** Non-treated non-hyperlipidemic rats (negative control) group. **(B)** Hyperlipidemic non treated rats (positive control). **(C)** Hyperlipidemic rats treated with fermented camel milk. **(D)** Hyperlipidemic rats treated with FCM fortified with 10% *Cordia myxa* fruit pulp. Scale bar, 50 μm, magnification, ×100.

The weight of white adipose tissue and liver, as well as the size of adipocyte cells, are reported in Table 8 and Figures 1A–D. The weight of adipose tissue and the size of adipocytes differed significantly (*p* ≤ 0.05) between the normal control and positive control groups, according to these findings. In contrast to the positive control group, FCM and FCM supplemented with CMP significantly decreased adipocyte size and adipose tissue weight (*p* < 0.05). In this work, for the first time, the combined effects of camel milk and CMP on obesity in a rat model given a high-fat diet were studied. These positive benefits were accompanied by a substantial decrease in the rats’ body weight growth. Adipocyte size decreased because the experimental groups had far less adipose tissue mass than the positive control group. Several earlier studies (72, 73) have demonstrated a decrease in adipocyte size and adipose tissue mass in FCM or *Cordia myxa* fruit. Our results are consistent with these findings.

**Table 8:** Effects of FCM supplemented with CMP on liver weights, adipose tissue and fat cell volume in hyperlipidemic rats.

Groups	Liver (g)	White adipose tissue (g)	Adipocyte size μm^3^
Group (1)	7.30 ± 0.76^d^	1.72 ± 0.48^d^	4.32 ± 0.46^d^
Group (2)	9.72 ± 0.84^a^	4.86 ± 0.76^a^	9.94 ± 0.62^a^
Group (3)	8.56 ± 0.52^b^	3.02 ± 0.44^b^	6.86 ± 0.58^b^
Group (4)	7.94 ± 0.66^c^	2.6 ± 0.52^c^	4.86 ± 0.42^c^
LSD	0.1414	0.2332	0.2975

Mean values of six rat’s ± SD. a, b, c…. of the small letters in the same column are significantly different at (*p* ≤ 0.05). LSD, Least significant difference.

### 3.9. Histopathological examination of liver

**Figure 2:**
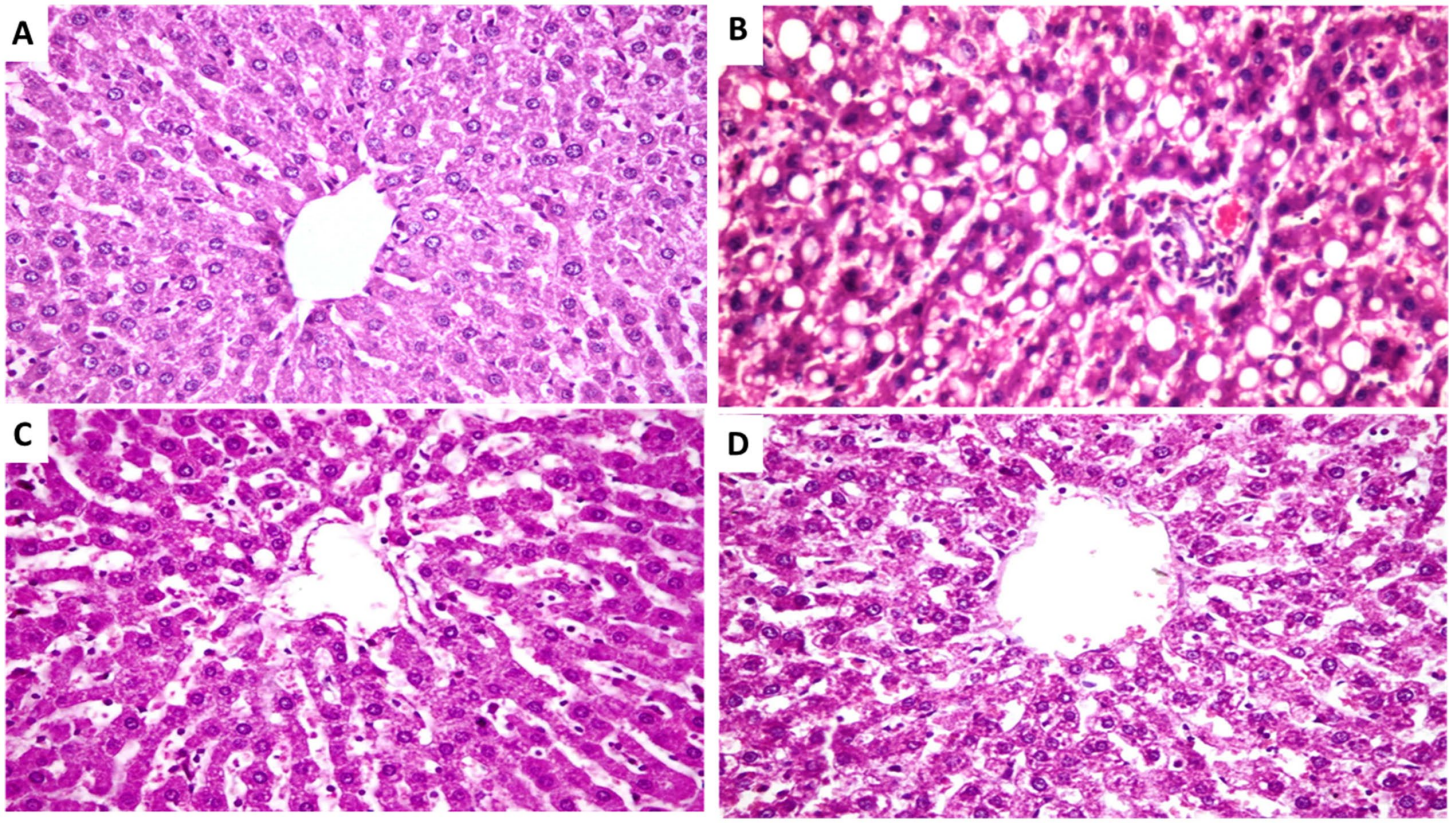
Photomicrograph from rats of experimental groups showing. **(A)** Liver showing normal hepatocytes and parenchyma belongs non-treated non-hyperlipidemic rats (negative control). **(B)** Liver of untreated group (positive control), showing severs and diffuses fatty degeneration in the form of circumscribed vacuolated hepatocytes among the hepatic parenchyma (arrows). **(C)** Liver of hyperlipidemic rats feeding on FCM, showing massively distributed of slightly vacuolated hepatocytes (arrows). **(D)** Liver of hyperlipidemic rats treated with FCM fortified with 10% *Cordia myxa* fruit pulp showing slightly dilated blood sinusoids in between the hepatic parenchyma (arrows). Hx&E stain, the bar size was ×400.

**Figure 3:**
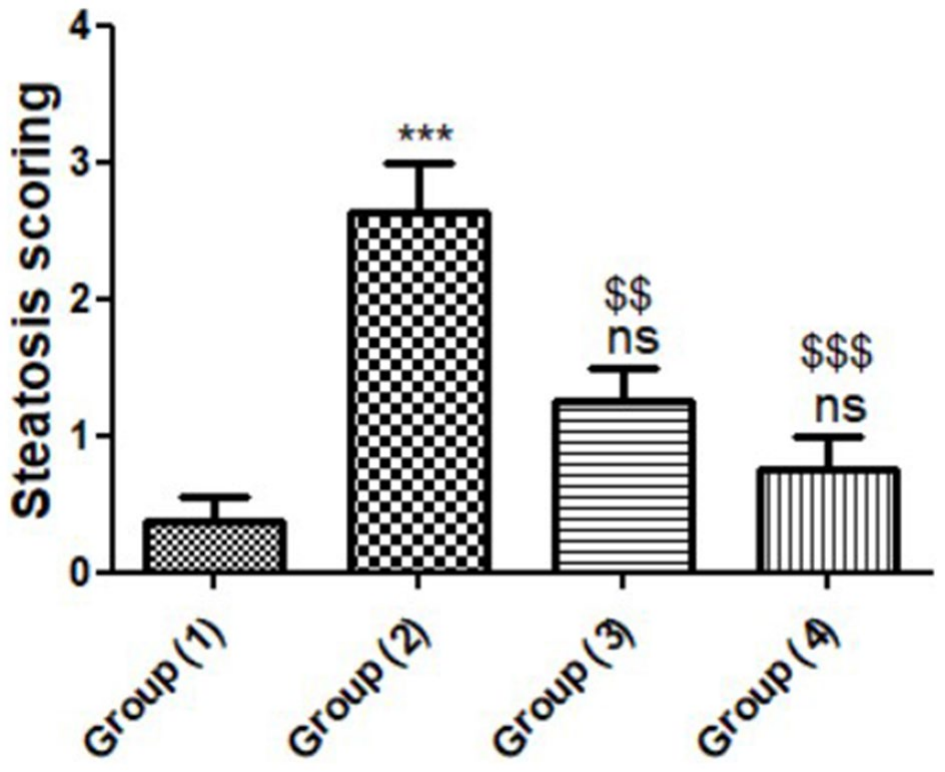
Histomorphometry graph showing semiquantitative assessment of steatosis scores recorded in hepatic tissue sections among the experimental groups. Data are expressed as means ± standard deviations. Significant differences vs. the control group (1) are marked by different asterisks, Group (2) vs. Groups (3&4) are marked by different $, through one-way ANOVA with Tukey’s *post hoc* test: ^$$^*p* ≤ 0.01, ***^,$$$^*p* ≤ 0.001.

Histological normality was shown by microscopic examination of the livers of rats from the normal control group (Figure 2A). The structure of the hepatic lobule liver of rats from the positive control group for the amitriptyline group showed severe and diffuse fatty degeneration in the form of circumscribed vacuolated hepatocytes among the hepatic parenchyma (Figure 2B). Meanwhile, the liver of rats from the treated group with FCM showed massively distributed slightly vacuolated hepatocytes, which is shown in Figure 2C. In addition, Figure 2D, found in the liver of rats from the treated group with FCM supplemented with CMP, showed slightly dilated blood sinusoids in between the hepatic parenchyma. The high concentration of bioactive compounds in *Cordia myxa* fruit has been demonstrated to boost liver function (25). In relation to histomorphometry (Figure 3), the semiquantitative assessment of steatosis scores recorded in hepatic tissue sections among the experimental groups showed a significant difference between the experimental groups. As depicted, the highest steatosis was reported among the positive control group (G2) while the lowed one with reported among the group of hyperlipidemic rats treated with FCM fortified with 10% *Cordia myxa* fruit pulp.

## 4. Conclusion

The current research indicated that *Cordia myxa* fruit and camel milk are efficient dietary supplements for avoiding overweight and obesity in rats when using fermented camel milk fortified with 10% of *Cordica mixa* fruit pulp at a rate of 10 g/by decreasing adipocyte size, weight gain, and blood lipid concentrations. Clearly, when camel milk was fortified with *Cordia myxa* fruit pulp, the antioxidant, chemical, sensory, and rheological characteristics of fermented camel milk (FCM) were improved. These improvements were proportionate to the level of fortification up to 10%, which added nutritional and health advantages to the final FCM. In comparison to hypercholesterolemic rats, consumption of FCM containing 10% *Cordia myxa* fruit pulp significantly decreased levels of MDA, TG, TC, ALT, AST, LDL, urea, and creatinine increased levels of total protein, HDL, and albumin. Clearly, fortification of camel milk with *Cordia myxa* fruit pulp enhanced the antioxidant, chemical, sensory, and rheological qualities of FCM. These improvements were proportionate to the level of fortification up to 10%, which added nutritional and health advantages to the final FCM product. It is advised that more research be conducted to investigate the benefits of incorporating *Cordia myxa* fruit into different dairy products. Furthermore, conducting further research on gene expression and protein dosage of transcription factors, enzymes related to lipogenesis and lipolysis, as well as hormonal measurements is highly recommended.

## Data availability statement

The original contributions presented in the study are included in the article/supplementary material, further inquiries can be directed to the corresponding author.

## Ethics statement

The animal study was reviewed and approved by the Faculty of Science, Tanta University and the Institutional Review Board Number IACUC-SCI-TU-0300.

## Author contributions

EA, MS, EAE-S, and MAAH were involved in the conception of the research idea and methodology design, supervision, performed data analysis and interpretation, and prepared the manuscript for publication and revision. ER-Á, MAH, ND, ME-K, AA, and EE were involved in methodology and drafted and prepared the manuscript for publication and revision. All authors have read and agreed to the published version of the manuscript.

## Funding

This study was supported by Princess Nourah bint Abdulrahman University Researchers Supporting Project number (PNURSP2023R23), Princess Nourah bint Abdulrahman University, Riyadh, Saudi Arabia.

## Conflict of interest

The authors declare that the research was conducted in the absence of any commercial or financial relationships that could be construed as a potential conflict of interest.

## Publisher’s note

All claims expressed in this article are solely those of the authors and do not necessarily represent those of their affiliated organizations, or those of the publisher, the editors and the reviewers. Any product that may be evaluated in this article, or claim that may be made by its manufacturer, is not guaranteed or endorsed by the publisher.
